# The Effect of DNA Topology on Observed Rates of R-Loop Formation and DNA Strand Cleavage by CRISPR Cas12a

**DOI:** 10.3390/genes10020169

**Published:** 2019-02-22

**Authors:** Kara van Aelst, Carlos J. Martínez-Santiago, Stephen J. Cross, Mark D. Szczelkun

**Affiliations:** 1 School of Biochemistry, Faculty of Life Sciences, University of Bristol, Bristol BS8 1TD, UK; kara.vanaelst@bristol.ac.uk; 2 DNA-Protein Interactions Unit, School of Biochemistry, Faculty of Life Sciences, University of Bristol, Bristol BS8 1TD, UK; carlosj.martinez-santiago@bristol.ac.uk; 3 Wolfson Bioimaging Facility, Faculty of Life Sciences, University of Bristol, Bristol BS8 1TD, UK; stephen.cross@bristol.ac.uk

**Keywords:** rapid reaction kinetics, endonuclease mechanism, DNA topology

## Abstract

Here we explored the mechanism of R-loop formation and DNA cleavage by type V CRISPR Cas12a (formerly known as Cpf1). We first used a single-molecule magnetic tweezers (MT) assay to show that R-loop formation by *Lachnospiraceae* bacterium ND2006 Cas12a is significantly enhanced by negative DNA supercoiling, as observed previously with *Streptococcus thermophilus* DGCC7710 CRISPR3 Cas9. Consistent with the MT data, the apparent rate of cleavage of supercoiled plasmid DNA was observed to be >50-fold faster than the apparent rates for linear DNA or nicked circular DNA because of topology-dependent differences in R-loop formation kinetics. Taking the differences into account, the cleavage data for all substrates can be fitted with the same apparent rate constants for the two strand-cleavage steps, with the first event >15-fold faster than the second. By independently following the ensemble cleavage of the non-target strand (NTS) and target strand (TS), we could show that the faster rate is due to NTS cleavage, the slower rate due to TS cleavage, as expected from previous studies.

## 1. Introduction

CRISPR (clustered regularly interspersed short palindromic repeats)-Cas (CRISPR-associated) systems evolved to defend microbes against bacteriophages and have been classified into different types based on their *cas* genes [1,2]. Due to their RNA-based programmable DNA-targeting capability, the type-II Cas9 effector nucleases have been widely adapted as tools for gene editing, and beyond [3]. More recently, the type V Cas12a effectors (formerly known as Cpf1) have also been shown to be active for gene editing [4,5]. The unique properties of Cas12a paralogues mean that, for many applications, they could become the gene editing enzyme of choice. Despite high-resolution crystal and electron microscopy (EM) structures, and further rapid progress, our knowledge of Cas12a is rudimentary [6]. Here we sought to understand the nuclease mechanism of *Lachnospiraceae* bacterium ND2006 Cas12a (LbCas12a) by examining the kinetics of DNA cleavage and the effect of DNA topology on the observed rates.

**Figure 1:**
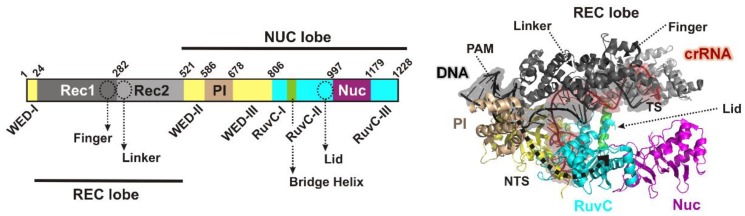
Domains and ternary structure of *Lachnospiraceae* bacterium ND2006 Cas12a (PDB: 5xus, TTTA PAM) [11]. Locations of “finger”, “linker” and “lid” from Stella et al. [8]; note that the “lid” is not resolved in PDB: 5xus. The putative path of the non-target strand (NTS) is shown on the structure as a thick dotted line, with the arrowhead pointing towards the RuvC active site.

Activation of the Cas12a nuclease activity requires R-loop formation between the CRISPR RNA (crRNA) and the DNA protospacer sequences [7,8,9,10]. A Cas12a-crRNA binary complex first binds DNA though interaction between a T-rich Protospacer Adjacent Motif (PAM, 5′-TTTV-3′, where V = A/C/G) [11], and a flexible pocket formed by the wedge (WED), REC1 and the PAM-interacting (PI) domains [7,11] (Figure 1). PAM distortion leads to ATP-independent stand separation and the DNA target strand (TS) forms a heteroduplex with the pre-structured 3′ end of the crRNA spacer sequence (the “seed”) [4,7,8,12], displacing the non-target strand (NTS). A 20 bp R-loop then propagates by dsDNA unzipping and pseudo A-form RNA hybridization, triggering DNA cleavage with some variability in the precise cut sites [4,13]. How Cas12a generates a dsDNA break has been a matter of some debate, with recent breakthroughs that help to clarify our understanding of the mechanism.

For Cas9, there are separate, classifiable nuclease domains, RuvC and HNH, which target the NTS and TS, respectively [14,15,16]. The HNH domain cleaves DNA faster than RuvC but it has been suggested that the conformational activation of the HNH domain controls the overall timing of DNA cleavage [17,18]. A classifiable RuvC domain is present in Cas12a, but a second nuclease domain was not identified from sequence/structure prediction alone [4,19]. An unclassified domain (Nuc, Figure 1) was suggested as the second nuclease on the basis that mutations produced DNA nicking [10]. Since RuvC mutations prevented any cleavage [10], an ordered strand-cleavage mechanism was proposed where the RuvC must act first and only then can Nuc carry out the second strand cleavage. Other groups argued that Nuc lacks identifiable catalytic residues and demonstrated that equivalent mutants still generated dsDNA cleavage [7]. The alternative suggestion is that Nuc regulates access to the RuvC active site which cuts both strands [6,8]. Structures of the related type V enzyme Cas12b [20,21], were also more consistent with Nuc acting in a noncatalytic role.

Closure of the Cas12a lobes moves the PI, REC1 and REC2 domains, exposing the RuvC nuclease and first guiding the displaced NTS towards RuvC [6,8,9,22,23], although none of the structures showed the DNA engaged with the active site. Stella et al. have recently identified a series of conformation checkpoints that couple R-loop propagation to nuclease activation [8]: Firstly a loop connecting REC1 and REC2 lobes (the “linker”, Figure 1) interacts with the 5th to 7th nucleotides of the crRNA as the R-loop forms; secondly, a loop (the “lid”, Figure 1) changes conformation, breaks contacts with the catalytic side chains of the RuvC nuclease, and interacts with the 8th to 11th nucleotides of the crRNA; and thirdly, a helix in the REC1 lobe (the “finger”, Figure 1) moves to interact with the 15th to 17th nucleotides of the crRNA. A requirement for more than 17 bp of hybrid to activate cleavage has been measured elsewhere [5,7,24].

Once activated, the exposed nuclease can now bind ssDNA, with the NTS being the most closely located (Figure 1). Accordingly, several studies have demonstrated a difference in the kinetics of NTS and TS cleavage. Jeon et al., (2018) used single-molecule fluorescence to identify distinct Fluorescence Resonance Energy Transfer (FRET) states that followed a sequential order during DNA cleavage by *Acidaminococcus* sp. Cas12a (AsCas12a) [25]. By using 51 bp oligoduplexes pre-nicked in either the NTS or TS, they assigned FRET states to NTS or TS cleavage events. The FRET transition profiles were then consistent with NTS cleavage before TS cleavage. But the lifetime of the states appeared similar (36 s and 58 s at 37 °C). In an extensive kinetic study, Strohkendl et al. followed NTS and TS cleavage by AsCas12a in separate reactions by individual radiolabeling of each strand of a 57 bp oligoduplex [26]. They determined maximal cleavage rate constants of 0.05 s^−1^ and 0.005 s^−1^ for the NTS and TS, respectively (at 25 °C), consistent with NTS cleavage preceding TS cleavage. Stella et al. [8] measured individual strand cleavage rates using labelled oligoduplexes. An NTS cleavage rate of ~0.007 s^−1^ (at 37 °C) was measured using a partially double-strand duplex where the NTS spacer was 24 nt but the TS spacer was 20 nt. A TS cleavage rate of ~0.0009 s^−1^ (at 37 °C) was measured using a partially double-strand duplex where the TS spacer was 20 nt but the TS spacer was only 14 nt. Again, the data is consistent with the NTS being cleaved before the TS. We note however that other studies used labelled DNA to follow NTS and TS cleavage but did not observe measurable differences in product appearance.

The activated open nuclease may accept the NTS more readily because of its orientation and location, with the TS only entering more slowly following a conformational transition (i.e., there isn’t a strict sequential order per se) [8]. This idea is consistent with the observation of *in trans* cleavage of bystander nonspecific ssDNA that is catalyzed by Cas12a upon recognition of a TS strand [22,23]. Alternatively, the single RuvC-Nuc nuclease might transition sequentially between the NTS and TS in a strictly ordered fashion, but other DNA could access the site at any point. 

Many studies of CRISPR Cas effectors have utilized short oligoduplexes as convenient substrates to monitor nucleic acid interactions and nuclease activity. However, using a single-molecule magnetic tweezers (MT) assay, we showed with both type II Cas9 and type I Cascade that on topologically-constrained linear DNA, negative supercoiling accelerates R-loop formation rates [27]. Due to the low/zero negative torque on unconstrained linear DNA, R-loop formation may become rate-limiting relative to other steps. Additionally, short oligoduplexes may become destabilized during R-loop formation, leading to fraying of DNA ends. This may favor R-loop formation in the absence of topology but may not mimic the situation in a genomic context where protospacers are away from free ends.

To address the torque-dependence of R-loop formation and DNA cleavage by LbCas12a, we first used the previously-described MT assay [27] to follow R-loop formation by LbCas12a in real time. The data shows that LbCas12a is torque-dependent, as expected, but less torque-stable than Cas9 or Cascade. By comparing cleavage kinetics on plasmid, nicked and linear DNA, we show that the microscopic DNA cleavage rate constants for the first cleavage event is >15-fold faster than the second, and that the observed cleavage of nicked and linear DNA were slower by at least 50-fold compared to negatively-supercoiled DNA. This can be explained by R-loop formation being rate-limiting on unconstrained linear DNA relative to cleavage. By labelling the TS and NTS strands of the plasmid-length linear DNA and comparing the data to simulations using the measured first and second strand rate constants, we could show that the more rapid cleavage rate is due to NTS cleavage. However, we could not distinguish between strict sequential and random models from the kinetics alone. We suggest that future studies of Cas12a (and other CRISPR-Cas effector nucleases) should always consider the effect of R-loop formation kinetics on the observed rates of downstream events including domain motions and strand cleavage events.

## 2. Materials and Methods

### 2.1. Protein

*Saccharomyces cerevisiae* Ubiquitin-like-specific protease 1 (SUMO-protease ULP1) was purified using pFGET19_Ulp1 and the published protocol [28]. A synthetic gene for LbCas12a, codon-optimized for *Escherichia coli* expression, was supplied by IDT, and cloned into pE-SUMO(Kan) (LifeSensors Inc, Malvern, PA, USA) using NEBuilder^®^ HiFi DNA Assembly (New England Biolabs, Ipswich, MA, USA). To express the protein, *E. coli* Tuner (DE3) were transformed with pE-SUMO(Kan)-Cas12a. Following overnight growth at 37 °C on LB-agar (kanamycin, 50 μg/mL), a single colony was used to inoculate 5 mL LB (kanamycin, 50 μg/mL). Following overnight growth at 37 °C, the 5 mL culture was used to inoculate 500 mL LB (kanamycin, 50 μg/mL), and the cells grown in a 2.5 L flask at 37 °C and 250 rpm shaking. At an OD_600_ of ~0.6–0.7, isopropyl β-D-1-thiogalactopyranoside was added to 1 mM, and cell continued overnight at 16 °C and 250 rpm shaking.

Cells were harvested, resuspended in 25 mL of Buffer A [50 mM Tris-Cl (pH 8.0), 500 mM NaCl, 5 mM MgCl_2_, 0.5 mM EDTA, 1 mM β-mercaptoethanol and EDTA-free protease inhibitor tablets according to the manufacturer’s instructions (Roche, Welwyn Garden City, UK)] and lysed by sonication. The cell extract was clarified by centrifugation at 100,736 *g* for 40 min. The supernatant was dialyzed for >1 hr at 4 °C against Buffer B [50 mM Tris-Cl (pH 8.0), 500 mM NaCl, 1 mM β-mercaptoethanol, 30 mM imidazole] using 10,000 Da cut-off Snake Skin dialysis tubing. The dialyzed sample was 0.45 µm filtered and loaded onto a 5-mL HisTrap column (GE Healthcare UK Ltd, Little Chalfont, UK), equilibrated in Buffer B. Bound proteins were eluted with a linear gradient of Imidazole in Buffer B (30–500 mM, 100 mL). Fractions containing Cas12a were pooled and dialyzed for 1 hr at 4 °C against two changes of Buffer C [50 mM Tris-Cl (pH 8.0), 200 mM NaCl, 1 mM β-mercaptoethanol] using 10,000 Da cut-off Snake Skin dialysis tubing. Ulp1 was added to the dialysis tubing and incubated overnight at 4 °C in fresh Buffer C.

NaCl and imidazole were added to 500 mM and 30 mM, respectively, and the dialyzed sample 0.45 μm filtered and loaded onto a 5-mL HisTrap column (GE Healthcare UK Ltd), equilibrated in Buffer B. The flow-through containing Cas12a was collected and the Sumo-tag and ULP1 protease eluted separately using Buffer B supplemented to 500 mM imidazole. Cas12a was concentrated and equilibrated into Buffer C using 50 kDa cut-off centrifugal filter units (Millipore, MA, USA). For storage at −20 °C, glycerol was added to 50% (*v/v*).

### 2.2. crRNA and Ribonucleoprotein Complex Assembly

The crRNA was produced by in vitro transcription. The oligodeoxyribonucleotides 5′-GAAATTAATACGACTCACTATCGGG-3′ and 5′-AGCTCGAATTGAAATTCTAAACGCATCTACACTTAGTAGAAATTCCCTATAGTGAGTCGTATTAATTTC-3′ (Eurofins genomics) were annealed at 40 μM in 50 mM NaCl by heating for 5 min at 95 °C followed by slow-cooling to room temperature (RT). This oligoduplex was then used in the HiScribe T7 high yield RNA synthesis kit (New England Biolabs) according to the manufacturer’s protocol. The samples were subjected to the optional DNase I treatment, and the crRNA purified using an RNA Clean & Concentrator (Zymo Research, Irvine, CA, USA). RNA concentrations were determined from UV absorbance at 260 nm. To assemble the ribonucleoprotein complex, 500 nM Cas12a and 500 nM crRNA were incubated in buffer SB [10 mM Tris (pH 7.5), 100 mM NaCl, 1 EDTA, 0.1 mM DTT, 5 μg/mL BSA] with RNase inhibitor (1U/ μL SUPERase in, Invitrogen, Carlsbad, CA, USA) for 1 hr at 37 °C, and the assembled complex used immediately.

### 2.3. DNA Substrates

For the MT assay, the plasmid pSP1 was used to prepare the biotin- and digoxigenin-labelled linear DNA substrate (see main text), as described previously [27,29]. For the supercoiled DNA used in the cleavage assays, *E. coli* Top10 (Invitrogen) or XL10-Gold (Stratagene, San Diego, CA, USA) were transformed with pSP1 [27], grown in M9 minimal medium supplemented with 37 MBq/l [^3^H-methyl] thymidine, and the DNA extracted using commercial protocols (Qiagen, Hilden, Germany) or by density gradient centrifugation in CsCl-ethidium bromide [30]. Open Circle or LIN1 DNA substrates were generated by incubating ^3^H-labelled pSP1 with 0.5 U/μl of BspQI or Nt.BspQI in NEBuffer 3.1 (New England Biolabs) for 1 hr at 50 °C. The DNA was purified by phenol/chloroform and chloroform extraction followed by ethanol precipitation. DNA concentrations were determined from UV absorbance at 260 nm, assuming that an optical density of 1 corresponds to 50 μg/mL DNA and a molecular weight of 6.6 × 10^5^ Da/kbp. On the linear substrate, the PAM sequence is 258 and 1853 bp from the free DNA ends.

For the strand-specificity experiments, PCR was used to generate a 2118 bp linear pSP1 fragment with the forward primer 5′-GCGTAAGTCTCGAGAACTAGTTCCGTAAGATGCTTTTCTGTGACT-3′ and the reverse primer 5′-GCGTAAGTGCGGCCGCTTCGTTCCACTGAGCGTCAGA-3′. To label the TS or NTS, the forward or reverse primer, respectively, was first 5′-labelled with ^32^P using T4-polynucleotide kinase. The PCR reactions were purified using a QIAquick PCR purification kit (Qiagen) and the DNA concentrations determined as above. As a marker, a commercial 1 kb DNA Ladder (New England Biolabs) was labelled with ^32^P using T4-polynucleotide kinase.

### 2.4. Magnetic Tweezers Assay

Magnetic tweezers measurements were performed using a commercial PicoTwist microscope (Fleurieux sur L’Arbresle, France) equipped with a Jai CV-A10 GE camera (image acquisition at 60 Hz) [31]. DNA molecules were tethered to 1-μm MyOne paramagnetic beads (Invitrogen) and anchored in flow cells as previously described [27]. Suitable topologically-constrained DNA were identified from rotation curves and the rotational zero reference (*Rot*_0_) set from a rotation curve at 0.3 pN. R-loop reactions were analyzed in Buffer SB at 25 °C, using 5-nM LbCas12a-crRNA. The shift in *Rot*_0_ upon R-loop formation was estimated by comparing the slope of the rotation curves when an R-loop was trapped in negative torque with the equivalent slopes in the absence of enzyme. Each trace in a reference set of 10 rotation curves collected in the absence of enzyme was compared to each rotation curve when an R-loop was trapped in the presence of enzyme (a total of 22) by using a least squares method to iteratively fit the matched data sets at −4.5 to –6.0 turns to parallel lines. The 220 parametrized linear fits where used to estimate the shift in the rotation curves and a local average was obtained for each R-loop event. A global average of the shifts in *Rot*_0_ was subsequently obtained by parametrizing the distribution of the local averages to a Gaussian function by maximum likelihood estimation. To analyze the shift due to the R-loop that remains trapped at positive torque, the same principle was applied. In this case, each trace in a reference set of 18 rotation curves collected in the presence of enzyme where the R-loop had already dissociated spontaneously at lower positive torque was compared to each of 2 curves in which the R-loop remained bound at +3.0 to +5.0 turns, and the global average of the shift in *Rot*_0_ was obtained from the 36 fits without assumption about the statistical distribution of the data. Torque values were calculated using software described in Ref [27]. Errors in the mean R-loop formation and dissociation times were calculated as the standard error of an exponential distribution, by dividing the mean reaction time by the square root of the number of events [29].

### 2.5. DNA Cleavage Assays

In vitro DNA cleavage reactions contained 3 nM DNA substrate and 50 nM LbCas12a-crRNA in Buffer RB [10 mM Tris (pH 7.5), 100 mM NaCl, 10 mM MgCl_2_, 0.1 mM DTT, 5 μg/mL BSA] at 25 °C. Reactions were initiated by addition of Cas12a-crRNA. For the native 1.5% (*w*/*v*) agarose gel electrophoresis in 1× TAE buffer, reactions were quenched at the time points indicated by 0.5 volumes of STEB [0.1 M Tris (pH 7.5), 0.2 M EDTA, 40% (*w*/*v*) sucrose, 0.4 mg/mL bromophenol blue] and heated for 10 min at 80 °C. The percentage of DNA in each band per lane was ascertained by scintillation counting [30]. Note that the smaller product of LIN1 cleavage was not clearly resolved on the gel. For alkaline denaturing 2% (*w*/*v*) agarose gel electrophoresis in 50 mM NaOH, 1 mM EDTA, reactions were quenched at the time points indicated by 0.5 volumes of Alkaline Buffer [300 mM NaOH, 6 mM EDTA, 18% (*w*/*v*) Ficoll 400, 0.1% (*w*/*v*) Bromocresol green and 0.1% (*w*/*v*) Xylene Cyanol] and heated for 10 min at 80 °C. Following electrophoresis, the gels were neutralized for 1 h in 500 mM Tris-HCl (pH 8), compressed for one hour, dried under vacuum for one hour, and a 16-bit densitometric phosphor screen scan analyzed using the 1D gel analysis software of ImageQuant (GE Healthcare). DNA fitting and simulations used numerical integration in Berkeley Madonna (www.berkeleymadonna.com). For the fits, averages and errors were calculated from the values returned from individual datasets.

## 3. Results

**Figure 2:**
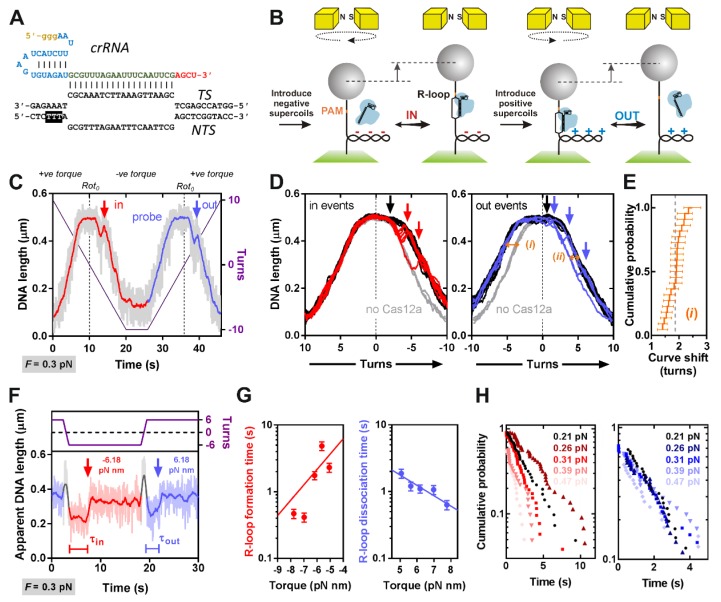
Measurement of R-loop formation by LbCas12a using a magnetic tweezers assay. (**A**) DNA protospacer sequence (black) and CRISPR RNA (crRNA) showing the G-residues from in vitro transcription (brown), the pseudoknot (blue) and spacer (red). (**B**) Principle of the MT assay. See main text. (**C**) R-loop cycling experiment (1 turn s^−1^) in the presence of 5 nM Cas12a:crRNA. Raw DNA length taken at 60 Hz (grey). Data smoothed by a 1 Hz moving average (dark colors). DNA is negatively supercoiled at 0.3 pN (red) to induce R-loop formation (in), followed by positive supercoiling to probe R-loop formation (blue), resulting in R-loop dissociation (out). *Rot*_0_ are points where DNA turns are zero. (**D**) Overlay of R-loop cycles (*N* = 22) for negative supercoiling (in events) and positive supercoiling (out events). Cycles without Cas12a are in grey. Data was smoothed by a 1 Hz moving average. (*i*) and (*ii*) show rotation curve shifts due to captured R-loops. (**E**) Rotation curve shift due to R-loop events (*i*). Average = 1.87 ± 0.27 turns (errors = SD). (**F**) Examples of repetitive R-loop formation cycling (at 10 turns s^−1^) to measure R-loop formation times. Raw and 1 Hz smoothed data are shown. (**G**) Mean R-loop formation/dissociation times and standard error (*N* = 40 to 52) as a function of torque [29]. Solid lines are fits to Equation (1) (Table 1) [27]. (**H**) Inverted cumulative probability over time for R-loop formation (left) and dissociation (right) used to calculate mean times in panel F.

A synthetic, codon-optimized gene for LbCas12a was inserted into pE-SUMO(Kan) using NEBuilder^®^ HiFi DNA Assembly (Section 2). Over-expressed protein was purified by Ni-column chromatography and the SUMO tag removed using SUMO Protease 1 (Ulp1) [28]. This liberates the full-length protein (1228 aa) without any tag. As a protospacer target we used the sequence in Figure 2A from pSP1 [27], with the corresponding crRNA synthesized using in vitro transcription (Section 2). The final four 3′ spacer nucleotides of the crRNA are complementary to the DNA protospacer but are not expected to participate in R-loop formation based on the structures. LbCas12a-crRNA complexes were formed by incubation at equimolar concentrations for one hour. All reactions were at a temperature of 25 °C.

### 3.1. Measurement of the R-Loop Formation Kinetics of LbCas12a Using a Magnetic Tweezers Assay

The principle of the MT assay is illustrated in Figure 2B (Section 2) [27,29]. In brief, a 2-kbp linear DNA substrate with the PAM-protospacer centrally located was ligated to 1-kbp DNA handles and tethered in a flow cell between the surface of a coverslip and a 1-μm streptavidin-coated paramagnetic particle. Using a commercial MT instrument (PicoTwist) [31], a pair of permanent magnets above the flow cell can be translated vertically to change the stretching force on the DNA and rotated to change the DNA twist. The position of the bead can be monitored in the vertical *z*-axis at 60 Hz, to give the apparent DNA length. Any change in DNA structure produced by enzyme action can then be observed from changes in the apparent DNA length. To prevent DNA cleavage, an EDTA reaction buffer was used to exclude any residual Mg^2+^ ions.

To favor R-loop formation in the presence of Cas12a-crRNA, the DNA is stretched with 0.21–0.47 pN vertical force and mechanically unwound (Figure 2B). The negative torque conditions favor reactions that unwind DNA. Under low force conditions, changes in DNA twist are converted to changes in writhe and negative plectonemic supercoils are formed, shortening the apparent DNA length [32]. Formation of an R-loop by Cas12a requires 20 bp of DNA unwinding [7,10], reducing the DNA twist. Since the tethered DNA is topologically-constrained, the linking number must remain constant so a compensatory increase in DNA twist occurs, reducing the negative supercoils, and increasing the apparent DNA length. The size of the length change is proportional to R-loop size. At the forces and number of turns used here, the torque is constant between the R-loop “out” and “in” states.

For a reaction in dynamic equilibrium, collapse of the R-loop will produce DNA rewinding and a corresponding reduction in apparent DNA length. However, we found with type I and II Cas effectors that R-loop formation was irreversible at negative torque [27]. To measure an off-rate, the magnets can be mechanically rotated to turn the particle and overwind the DNA, generating positive supercoils and a corresponding reduction in apparent DNA length. Positive torque favors DNA rewinding and thus R-loop collapse. The compensatory decrease in DNA twist reduces the number of positive supercoils and the apparent DNA length increases. The cycle can then be repeated. If the magnet rotation is sufficiently rapid so that the change in supercoiling can happen before dissociation/association, on/off rates can be calculated. The torque can be changed by changing the stretching force on the DNA [27].

An example R-loop cycling experiment with slow magnet turns (1 s^−1^) is shown in Figure 2C. At low negative torque, an R-loop forms as observed by the increase in apparent DNA length (red arrow). To probe R-loop dissociation, the DNA was rewound to produce positive supercoils, and the R-loop dissociated (blue arrow). In many cases (e.g., 15/22 in Figure 2D), Cas12a formed an R-loop instantaneously at −1 to −2 turns; although this does not result in a noticeable change in DNA length, R-loop formation can be observed by the shift in the left side of the probe curve relative to the no enzyme control (compare black and grey lines). In some cases (e.g., 7/22 in Figure 2D), R-loop formation required longer times and more negative supercoiling (red lines). In these cases, the jump in DNA length can be observed (red arrows). The shift in the R-loop curves (Section 2) was 1.87 ± 0.27 turns for the R-loops captured in the negative torque regime (events (*i*) in Figure 2D), very close to the expected 1.9 turns for a 20 bp R-loop (assuming 10.5 bp per turn) (Figure 2E).

In most cases, R-loop dissociation was spontaneous at +1 to +2 turns (e.g., 18/22 events in Figure 2D). Three events in Figure 2D show a jump in DNA length, two of those most clearly at >+2 turns (blue arrows). A fourth event appeared substantially more stable, surviving until >+10 turns. For the events where the LbCas12a R-loop survived into the positive torque regime, the shift in the R-loop curves (events *ii* in Figure 2D) could be estimated as 1.12 ± 0.12 turns, smaller than the 1.8 7± 0.27 turns trapped by the R-loop in the negative torque regime (events *i*). At the force range used here, the relationship between DNA length and supercoiling is symmetrical at both negative and positive turns (Figure 2D) [33], so one would expect the changes in DNA length to be comparable for formation and dissociation events. An explanation is that R-loop dissociation is occurring in 2 sub-steps, with the first sub-step happening spontaneously at low positive torque where a clear jump in DNA length is hard to observe, and the second sub-step being more torque stable. It is the second step that is observed as clear DNA length change events in Figure 2D (blue arrows).

In order to measure the kinetics of R-loop formation and dissociation, we used more rapid magnet revolutions (10 turns s^−1^) (example in Figure 2F). From repeated R-loop cycles, we obtained the formation and dissociation times as a function of torque (Figure 2G,H). R-loop formation occurred in a single measurable step, with a shallow torque dependence and kinetics similar to Cas9. R-loop dissociation also occurred in a single measurable step; we did not observe any sub-steps indicative of two sequential dissociation steps. Because the R-loop lifetimes were short relative to experimental noise and camera frame rate, it was difficult to measure the change in DNA length for each event and to confirm if they were smaller than the DNA length changes for R-loop formation, as suggested by the data in Figure 2D.

The product of a two-step process can be fitted with a simple analytical solution [34]; for a scheme where the rates are equal, this will result in a noticeable lag time in product appearance. However, we could not distinguish a lag in the R-loop dissociation time distributions. Based on the torque instability seen at slow rotations in Figure 2D, the first dissociation event may have a short lifetime at low torque, even occurring during the more rapid particle rotation in Figure 2F. Where a first step in a two-step process is much faster than the second step, the curve will tend towards a single exponential, which we used as an estimate of the dissociation time in Figure 2H. Therefore, the dissociation time constants most likely reflect the second dissociation event only. They nonetheless show a shallow torque dependence with kinetics similar to Cas9.

The relationship between the R-loop kinetics and the applied torque can described by an Arrhenius-like exponential relationship (Figure 2G) [27]:

(1) $$\tau_{in/out}\left(\Gamma \right)=\tau_{in/out}\left(\Gamma =0\right)e^{\Gamma 2\pi \Delta N_{in/out}^{*}/k_{B}T}$$

where *Γ* is torque, *τ*_in/out_ (*Γ*) is the R-loop formation (in) or dissociation (out) time at a given torque, *τ*_in/out_ (*Γ* = 0) is a pre-exponential factor that estimates the R-loop formation/dissociation time at zero torque, and ∆*N* *_in/out_ is the distance of the initial state to the transition state barrier (in turns). The fitted values from Figure 2G are shown in Table 1. Note that pre-exponential factors are subject to additional inaccuracy since the measurements are at elevated torque levels, and the R-loop formation times also show high scatter. To determine the equilibrium constant for R-loop formation, we would need to determine the transition time for the first dissociation step, which was not possible under our conditions.

### 3.2. Negative DNA Supercoiling Supports a Faster Apparent Rate of DNA Cleavage by LbCas12a

R-loop formation by LbCas12a in the absence of Mg^2+^ ions is accelerated by negative torque (Figure 2, Table 1). Since an R-loop is necessary to activate nuclease activity, the kinetics of DNA cleavage might be indirectly influenced by the topology of the substrate. To assess this, we compared ^3^H-labelled negatively supercoiled plasmid (SC), pre-nicked DNA (open circle, OC) and linear DNA (LIN1) substrates under pre-steady state conditions, with the substrate saturated with enzyme and the cleavage reactions representing a single turnover (Section 2). Plasmid DNA purified from *E. coli* has a superhelical density of σ ≈ −0.05, which corresponds to a negative torque of ~−7 pN nm [35,36]. Since this is in the same range as the data in Figure 2G, we would expect R-loop formation to occur within a few seconds on supercoiled plasmid DNA (assuming similar R-loop kinetics in the presence of Mg^2+^ ions). A pre-nicked DNA was generated from the plasmid by single-strand cleavage using Nt.BspQI at a site 258 bp from the 5′ end of the PAM. LIN1 was generated from the plasmid by double-strand cleavage using BspQI. The pre-nicked and linear DNA have effectively zero torque, so would be expected to have a slower R-loop formation time (e.g., taking >30 s, Table 1) that might limit the observed cleavage rates relative to a supercoiled plasmid.

**Figure 3:**
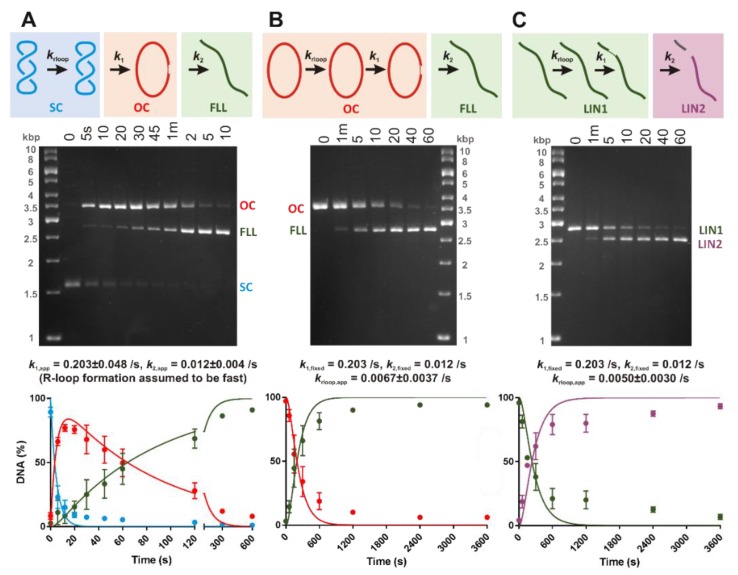
Apparent DNA cleavage rates are affected by substrate topology. Cleavage of 3 nM supercoiled (**A**), pre-nicked circular (**B**) and linear (**C**) substrates by 50 nM LbCas12a-crRNA. Diagrams represent the substrate, intermediate and product states: supercoiled (SC); nicked (open circle, OC); full length linear (FLL); linear substrate (LIN1); and products of linear DNA cleavage (LIN2). DNA species were separated by native agarose gel electrophoresis. (The OC and LIN1 data shown are from the same gel and thus share the same marker lane). The kinetic models were simultaneously fitted (solid lines) to each of three repeats and the rate constants averaged (Table 2, with SD errors). For panel A, *k*_rloop_ was fixed as infinitely fast. For panels (**B**) and (**C**), the *k*_1_ and *k*_2_ values were fixed using the fits in panel (**A**). Plotted points are the average of the 3 repeats, errors bars as SD.

Reactions were initiated by addition of saturating LbCas12a-crRNA complex to a DNA master mix (in a Mg^2+^ buffer), and aliquots quenched in EDTA at the time points indicated in Figure 3. DNA species were separated by native agarose gel electrophoresis, and the bands quantified by scintillation counting (Section 2). This pre-steady state method does not distinguish enzyme bound and free states, so reports DNA cleavage rates, not product release. Note that cleavage of the pre-nicked and LIN1 substrates was significantly slower than the supercoiled plasmid, so the reaction time points and *x*-axes differ between Figure 3A–C.

To extract kinetic constants, we fitted simultaneously the percentages of the reaction species to the corresponding kinetic models shown in the diagrams in Figure 3, where *k*_rloop_ is an apparent rate constant for R-loop formation (DNA binding assumed to be too fast to measure), and *k*_1_ and *k*_2_ are apparent rate constants for the first and second strand cleavage steps, respectively (Section 2) (Table 2). At this stage we consider these as macroscopic rate constants that may represent multiple pathways, depending on the cleavage scheme. Because we are not independently following cleavage of the NTS and TS, it is difficult to distinguish between these schemes at this stage (see below).

For SC cleavage, the fitted rate constant for the first strand cleavage was ~17-fold faster than the second strand cleavage, and correspondingly the peak maximum for the OC intermediate was ~80% (Figure 3, Table 2). This apparent difference in rates for the two cleavage steps is consistent with previous observations [26]. If we assume that the measured rate constants represent DNA cleavage steps, we could not extract a value for *k*_rloop_ from the fit as the reaction appeared faster than the lifetime of the first cleavage step (~5 s), so this constant was set as “infinitely fast” (Table 2). Relatively fast R-loop formation with negative supercoiling is consistent with the MT data in Figure 2G. An alternative interpretation of the data would be that the first strand cleavage is fast, and the observed rate constant is limited by the R-loop formation rate. This would give a time constant of ~5 s for R-loop formation, which is at the upper (slow) end of the times seen in Figure 2G.

For the pre-nicked and LIN1 substrates, the first strand nicking event cannot be observed on a native agarose gel as the substrate and intermediate have the same mobility (Figure 3B,C). We reasoned that if R-loop formation is torque-sensitive but the microscopic DNA cleavage rates were unaffected by topology, we could fix *k*_1_ and *k*_2_ in the fit using the values from the SC DNA (Figure 3A). The observed OC and LIN2 cleavage rates could then be fitted by floating *k*_rloop_. Using this approach we could satisfactorily fit the OC and LIN1 data (Figure 3B,C), and return a lifetime for R-loop formation in the absence of torque and presence of Mg^2+^ of 100–500 s (including the full error range) (Table 2). Therefore, the apparent DNA cleavage rate of LbCas12a is very sensitive to torque, as suggested by the MT assay data in Figure 2, Table 1.

### 3.3. LbCas12a Catalyses Sequential Cleavage of the Non-Target Strand Followed by the Target Strand

**Figure 4:**
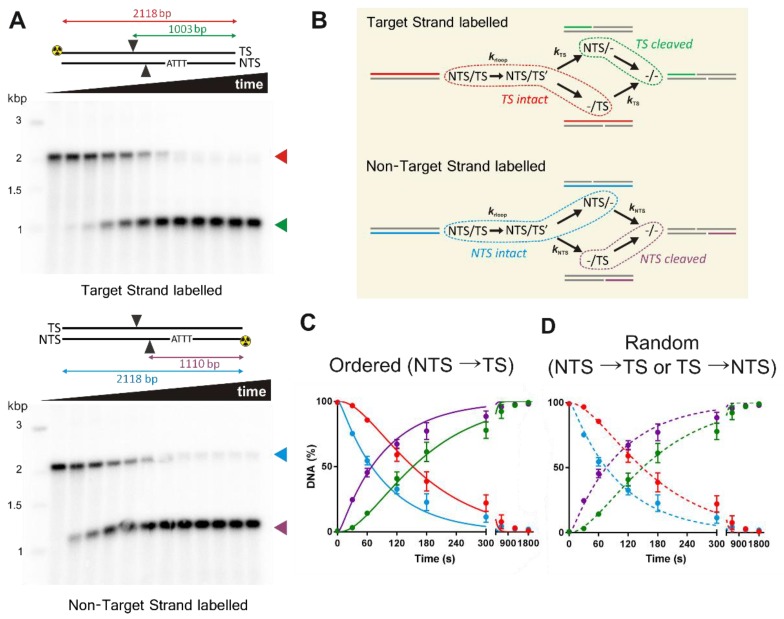
LbCas12a cleaves the non-target strand faster than the target strand. (**A**) Linear DNA substrates were ^32^P-labelled on either the TS (upper panel) or NTS (lower panel). The PAM, approximate positions of NTS and TS cleavage, and sizes of the intact and cut labelled-strands are indicated. DNA species were separated by alkaline denaturing agarose gel electrophoresis. Each lane represents the reaction following LbCas12a addition (left to right): 0, 0.5, 1, 2, 3, 5, 10, 20, 30, 40, 50 and 60 min. (**B**) Diagrams showing overlap between substrate, intermediates and products depending on the labelled strand. Dotted lines enclose those species which will have the same labelled strand identity on a denaturing gel (**C**) Ordered kinetic model and (**D**) random kinetic model, simulated (solid and dashed lines, respectively) using values in Table 2. NTS and TS data points in both panels came from separate gels, as in panel (**A**), and are the average from 3 repeat experiments, with error bars as SD.

Do the *k*_1_ and *k*_2_ rate constants from Figure 3 represent the expected rapid cleavage rate for the NTS followed by the slower rate for the TS, respectively? To follow the cleavage of individual strands, we used PCR to generate 2,518 bp linear substrates equivalent to those used in the MT assays (Figure 2). We 5′-labelled one or other primer with ^32^P so that cleavage of the NTS or TS strand could be followed in separate reactions (Figure 4A). Cleavage was measured as above except that the DNA species were separated by alkaline denaturing agarose gel electrophoresis, and the bands quantified by densitometry (Section 2). The gels show that the NTS is cleaved earlier than the TS, as expected from previous studies and the current model.

To determine whether the data in Figure 4A can match the rate constants determined in Figure 3C, we needed to analyze the data using a suitable minimal kinetic model (Figure 4B). Where the TS is labelled, the cleavage of the unlabeled NTS will not be observed using a denaturing gel. Similarly, where the NTS is labelled, cleavage of the unlabeled TS will not be observed. Taking this into account, we compared the data from both TS and NTS gels with a simulation of the complete kinetic pathway using different cleavage schemes. In an ordered scheme, these is an absolute defined order of cleavage, where the NTS is cleaved first and only then can the TS be cleaved. In Figure 4B only the lower pathway would be followed, and the TS intermediate will never be observed. Alternatively, the order of cleavage of the NTS or TS is a completely random choice of either upper or lower pathway, determined only by the relative rate constants.

In Figure 4C, we plot the quantified data from Figure 4A against a simulation of an ordered scheme where the NTS must be cleaved first followed by the TS. The NTS and TS strand cleavage rate constants were taken from the fits in Figure 3 (i.e., *k*_1_ and *k*_2_, respectively, Table 2). The model could accurately describe the data using the *k*_1_ and *k*_2_ values, but the R-loop formation rate needed to be ~twofold faster than *k*_rloop_ from Figure 3C (Table 2). In Figure 4D, we plot the quantified data from Figure 4A against a simulation of a random scheme using the same rate constant values. In this case we used *k*_1_ as the values for NTS cleavage as either a first or second step, and *k*_2_ as the values for TS cleavage as either a first or second step. Again, the model provides a good match to the strand-specific cleavage data.

## 4. Discussion

We measured R-loop formation for LbCas12a (Figure 1) using the MT assays previously applied to Cas9 and Cascade (Figure 2) [27]. Our data are consistent with an ~20 bp R-loop as observed in the crystal structures (although the rotational shifts measured in Figure 2D,E are an indirect measurement that can be altered by DNA wrapping/bending, [27]). The data is most similar to that observed with type II Cas9, which was strikingly less torque stable than type I Cascade. Both the rotation curves and kinetic data (Figure 2D,G) indicate that Cas12a is more torque sensitive than Cas9, with spontaneous R-loop formation and dissociation at low torque values. For *Streptococcus thermophilus* DGCC7710 CRISPR3 Cas9, the estimated transition time for R-loop dissociation at zero torque was ~130 s [27], suggesting it is >10-fold more stable than LbCas12a (Table 1). Cas12a torque-instability may be a benefit in preventing off target effects, as mismatches may have a greater destabilization effect. A survey of mismatch effects has been undertaken for linear DNA targets in vitro by Strohkendl et al. [26], as well as by others [4,7,24]. It would be interesting to determine whether the effects of mismatches are impacted by DNA supercoiling.

We observed a striking difference in the apparent DNA cleavage rates on negatively supercoiled plasmid DNA compared to topologically-unconstrained substrates (Figure 3). Since negative DNA supercoiling favors R-loop formation (Figure 2), this can be readily explained by DNA cleavage being rate-limited by R-loop formation in the absence of negative torque; R-loops will form within a second on negatively supercoiled DNA but could take tens to hundreds of seconds on linear or nicked DNA. Many previous studies of type V systems have used linear DNA substrates. Caution is needed when analyzing downstream events (e.g., cleavage kinetics) to ensure that the effect of R-loop formation rates is considered. Rates may also be affected by the position of the protospacer relative to DNA ends; being close to an end may allow more rapid R-loop formation due to capture of free ssDNA produced by thermal fraying and/or because the thermodynamics of strand separation are altered.

Consistent with other studies [8,26], we observed a more than 15-fold difference in the rate of cleavage of the NTS compared to the TS (Figure 3 and Figure 4, Table 2). Because of the differences in the first and second strand cleavage rates, it is difficult to distinguish between a strict ordered mechanism and a random mechanism based on our kinetics. A limitation of the approach in Figure 4 is that the labelled ssDNA can represent multiple species on the reaction pathway (Figure 4B). A potential solution is a substrate where the TS and NTS nicked intermediates can be resolved. One way to achieve this would be a self-complementary hairpin oligonucleotide substrate, with an analysis label (e.g., a fluorophore) within the ssDNA hairpin loop, and separation of the DNA species on a denaturing gel. For a staggered cut as produced by Cas12a, this would allow the resolution in a single reaction of labelled substrate, both intermediates, and product as fragments of different lengths. However, because of the 15-fold difference in strand cleavage rates observed here, and compounded by slow R-loop formation rate on relaxed DNA, one would need to be confident in distinguishing 0% TS nicked intermediate (for an NTS→TS ordered model) from ~0.2% maximum TS nicked intermediate (for a random model). Even if the R-loop formation rate could be accelerated, the difference in expected TS intermediate species would still only be 0% and 2%, respectively.

The slower observed rate constant for TS cleavage could be due to a slow conformational transition that is required to engage the TS in the RuvC active site [7,8]. The PAM-distal TS cleavage site is distant (>40 Å) from RuvC in the Cas12a structures (Figure 1). Therefore, significant movement of RuvC-Nuc and/or the DNA-RNA must be required. One option is that the TS region must unwind following NTS cleavage to expose ssDNA for cleavage and this moves into the active site (“DNA reeling”). Stella et al. suggest that the R-loop may partially unwind to facilitate this [8]. Alternatively, the RuvC-Nuc domains could move towards the distal TS cleavage site (i.e., closing the nuclease and Rec lobes). A conformation change step following NTS cleavage was not included in the consideration of our kinetic models. Without being able to measure the intermediate species, adding further parameters to the model would not have returned sensible fitted values. Alternative approaches, such as FRET or structural determination (e.g., [8,24]), will be required to reveal what movements in the protein and DNA/RNA are required.

## Figures and Tables

**Table 1:** Constants from fits of data in Figure 2G to Equation (1).

			Transition	Barrier Distance (∆*N* *) ^a^	*τ*_in_ (*Γ* = 0) ^a^	*τ*_out_ (*Γ* = 0) ^a^
MT Linear DNA	Figure 2G	EDTA	R-loop formation	+0.34 ± 0.04 turns	52 ± 17 s	n.a
MT Linear DNA	Figure 2G	EDTA	R-loop dissociation (second sub-step)	−0.23 ± 0.01 turns	n.a.	11 ± 1 s

^a^ Errors = SEM.

**Table 2:** Comparison of kinetic rate constants for R-loop formation and DNA cleavage.

			*Γ*	*k*_rloop,app_^a^	*k*_1,app_^a^	*k*_2,app_^a^
SC	Figure 3	Mg^2+^	~−7 pN nm	*fixed as* ≫ *k*_1,app_ (*τ*_in_ < 5 *s*)	0.203 ± 0.048 s^−1^	0.012 ± 0.004 s^−1^
OC	Figure 3	Mg^2+^	0 pN nm	0.0067 ± 0.0037 s^−1^ (*τ*_in_ ≈ 149 s)	fixed as 0.203 s^−1^	fixed as 0.012 s^−1^
LIN1	Figure 3	Mg^2+^	0 pN nm	0.0050 ± 0.0030 s^−1^ (*τ*_in_ ≈ 200 s)	fixed as 0.203 s^−1^	fixed as 0.012 s^−1^
LIN PCR	Figure 4C,D	Mg^2+^	0 pN nm	0.0098 s^−1^ (*τ*_in_ ≈ 102 s) ^b^	fixed as 0.203 s^−1^	fixed as 0.012 s^−1^

^a^ Errors = SD. ^b^ The same values were used for either the ordered or random simulation.
